# What enables good end of life care for people with dementia? A multi-method qualitative study with key stakeholders

**DOI:** 10.1186/s12877-018-0983-0

**Published:** 2018-12-04

**Authors:** Claire Bamford, Richard Lee, Emma McLellan, Marie Poole, Karen Harrison-Dening, Julian Hughes, Louise Robinson, Catherine Exley

**Affiliations:** 1 Institute of Health and Society, Newcastle University, 2nd Floor, Newcastle Biomedical Research Building, Campus for Ageing and Vitality, Newcastle upon Tyne, NE4 5PL UK; 2 Department of Social Work, Education and Community Wellbeing, Faculty of Health & Life Sciences, Northumbria University, Newcastle-upon-Tyne, UK; 3 Dementia UK, Second Floor Resource for London, 356 Holloway Road, London, N7 6PA UK; 4 Bristol Medical School, Population and Health Sciences, University of Bristol, Bristol, BS8 2PS UK; 5 Health and Life Sciences, Northumbria University, Room NB266, Northumberland Building, College Street, Newcastle upon Tyne, NE1 8ST UK

**Keywords:** Dementia, End of life care, Qualitative research, Family caregivers, Palliative care

## Abstract

**Background:**

People with advanced dementia often experience suboptimal end of life care (EoLC) with inadequate pain control, increased hospitalisation, and fewer palliative care interventions compared to those with cancer. Existing policy, guidance and recommendations are based largely on expert opinion because of a shortage of high quality, empirical research. Previous studies have tended to consider the views and experience of particular groups. Whilst providing important evidence, they do not take into account the diversity of perspectives of different stakeholders. The Supporting Excellence in End of life care in Dementia (SEED) programme involved multiple stakeholder groups and an integrative analysis to identify key components of good EoLC for people with dementia and to inform a new intervention.

**Methods:**

The views of national experts, service managers, frontline staff, people with dementia and family carers were explored using a range of qualitative methods (semi-structured interviews, focus groups, discussions and observations of routine care). The large dataset comprises 116 interviews, 12 focus groups and 256 h of observation. Each dataset was initially analysed thematically prior to an integrative analysis, which drew out key themes across stakeholder groups.

**Results:**

Through the integrative analysis seven key factors required for the delivery of good EoLC for people with dementia were identified: timely planning discussions; recognition of end of life and provision of supportive care; co-ordination of care; effective working relationships with primary care; managing hospitalisation; continuing care after death; and valuing staff and ongoing learning. These factors span the entire illness trajectory from planning at a relatively early stage in the illness to continuing care after death.

**Conclusions:**

This unique study has confirmed the relevance of much of the content of existing end of life frameworks to dementia. It has highlighted seven key areas that are particularly important in dementia care. The data are being used to develop an evidence-based intervention to support professionals to deliver better EoLC in dementia.

**Electronic supplementary material:**

The online version of this article (10.1186/s12877-018-0983-0) contains supplementary material, which is available to authorized users.

## Background

By 2050, it is estimated that around 131 million people worldwide will live with dementia [1]. In the United Kingdom (UK) dementia is the leading cause of death in older women [2]. Although there is international variation in place of death, overall few people with dementia die at home with around one third dying in acute hospitals [3, 4]. Concerns exist about the quality of end of life care (EoLC) in dementia, especially in comparison to people dying with cancer, with research demonstrating suboptimal symptom management [5–7]. Although some interventions have demonstrated that low symptom burden can be achieved for people with dementia at the end of life [8] other interventions have failed to improve pain management [9, 10]. Transitions at the end of life (e.g. from home to hospital) remain an issue in dementia, although there is some evidence that the situation may be improving in some countries [11] or for some patient groups, with one study finding that patients with severe dementia were less likely to be transferred between care settings in the last 3 months of life and less likely to die in hospital [12].

High quality, empirical research to inform evidence-based recommendations on the delivery of good EoLC for people with dementia has been lacking [13–15], although this is improving [16]. While European guidelines have been developed via a professional Delphi consensus [17], few national dementia strategies explicitly address palliative care [18]. Studies continue to identify a range of barriers to the delivery of optimal palliative care in dementia [19–22]. To attempt to understand the implementation gap, previous studies have explored the views and experiences of service users and providers, although people with dementia remain a neglected group [23]. While a number of studies have integrated the perspectives of two or more stakeholder groups [19, 24–27], few have included the perspectives of people with dementia, family carers, health and social care professionals, and the majority have focused on people with dementia living in care homes. Only a minority of these studies have used their findings to develop evidence-based interventions for implementation and evaluation in practice [14, 28].

This paper reports an integrative analysis of a series of qualitative studies which aimed to understand the factors that facilitate good EoLC in dementia in England from the perspectives of a range of stakeholders and direct observation of care. It explores the extent to which priorities are shared by national experts, service managers, frontline staff, family carers and people with dementia. The findings will be used to develop an intervention to improve EoLC in dementia (to be reported elsewhere).

## Methods

Data were collected between October 2013 and January 2016 as part of a research programme on supporting professionals to deliver good EoLC in dementia (https://research.ncl.ac.uk/seed/). We adopted a social constructivist perspective [29], meaning that we understand knowledge and beliefs to be co-constructed. This perspective enabled us to capture and critically explore multiple perspectives of EoLC for people with dementia, in order to demonstrate what was seen to be working well and for whom. The programme comprised a series of interrelated and cumulative phases of data collection with multiple stakeholders; findings from individual datasets have already been published [30–32]. In this paper, we report an integrative analysis which highlights similarities and differences in the perspectives and priorities of different stakeholder groups. The large dataset comprised a) interviews and focus groups with a range of stakeholders and b) comparative case studies of three services providing end of life care. An overview of study methods and participants is provided in Table 1. Full details of the methods for the interviews and focus groups, including participants and topic guides, have previously been published [30–32] and are summarised briefly here.

**Table 1:** Study participants

Stakeholders (notation used in quotations)	Inclusion criteria	Methods	Number of participants in interviews (focus groups)	Comparative case studies: number of participants in:	
				Interviews (focus groups)	Observation
National experts (NE) [30]	Professionals with academic and/or clinic expertise in dementia and/or EoLC; policy experts	Telephone and face-to-face semi-structured interviews	30 (0)	NA	NA
Service managers (SM) [31]	Professionals managing care homes, hospices and home care services for people with dementia; service development leads in such services	Telephone and face-to-face semi-structured interviews Observation	33 (0)	2 (0)	2
Frontline staff (FS) [31]	Care assistants, senior care assistants and nurses in services providing EoLC to people with dementia. (Some service managers and service development leads also took part in focus groups)	Focus groups and observation	0 (53)	16 (6)	76
People with dementia (PWD) [32]	People with dementia who had joined the Case Register for those willing to take part in research studies and those registered with Join Dementia Research. People with dementia resident in services participating in the comparative case studies	Individual face-to-face interviews following a Q-sort activity Observation	11 (0)	0 (0)	40
Family carers (BC – bereaved carers; CC – current carers) [32]	Bereaved and current carers of people with dementia who received support from participating services.	Face-to-face semi-structured interviews One focus group Observation	18 (4)	3 (0)	2
Health care professionals (HCP)	Professionals providing care to residents in comparative case study sites	Face to face semi-structured interviews Observation	NA	3 (0)	2

The interview and focus group data (collected by CB, RL, EM and MP) focused on accounts of EoLC in dementia and perceived barriers and facilitators to the delivery of such care. Topic guides were used to structure the discussion while allowing for the emergence of new ideas from participants. Interviews and focus groups were audio-recorded, transcribed in full and anonymised for analysis.

The comparative case studies aimed to increase our understanding of how EoLC for people with dementia and their families was enacted in real world practice. Three of the eight services which participated in the focus groups were purposively sampled in terms of perceived practice (‘usual’ or ‘good’ practice); type of provider (not-for-profit; privately owned); types of residents (older people with mental health difficulties; residential; nursing) and invited to take part in the comparative case studies (a brief description of each service is provided in Additional file 1). Researchers (CB, RL, EM, MP) undertook ethnographic observations [33] which involved observing the delivery of care, together with further informal discussions and semi-structured interviews to explore issues arising from observation in more depth. One focus group was carried out at one site towards the end of data collection to explore emerging issues from a range of perspectives in more depth than was possible during observation. Observations, informal discussions and reflections were recorded in anonymised fieldnotes; interviews were audio recorded and transcribed (with one exception where the participant did not wish to be recorded when details were recorded in contemporaneous notes).

In total, the studies included 259 participants (this is less than the sum of participants in Table 1 since some participants took part in more than one component of the study). Prior to data collection, written informed consent was obtained from all participants with capacity to consent for themselves and the opinion of a personal or nominated consultee sought for those people with dementia unable to consent for themselves [34].

### Analysis

The interview and focus group data were thematically analysed and details are reported elsewhere [30–32]. For the comparative case studies, the qualitative team (CB, CE, RL, EM and MP) read and reread a subset of purposively sampled transcripts and fieldnotes to identify key issues. Emergent themes were discussed in data workshops; we then iteratively reviewed new data items against the draft themes. Findings were discussed in further data workshops, with the themes being modified as needed until a final version of the codes was agreed. Because of the volume of data from the comparative case studies, not all data were coded in full; instead, remaining fieldnotes were reviewed to ensure that all relevant issues had been captured.

To understand how the themes and subthemes in each dataset related to one another, we then conducted an integrative analysis. This process was informed by memos and narrative summaries and facilitated by a process of visual mapping of themes and concepts. The integrative analysis involved reconceptualising and developing themes to reflect the nuances in the data from different stakeholders. The findings presented here are therefore distinct from the themes previously reported for individual stakeholder groups.

The studies were approved by Newcastle University (00665/2013 – for interviews with national experts) and the National Research Ethics Service Committee North East – Newcastle & North Tyneside 1 (13/NE/0335 – for interviews and focus groups with service managers, frontline staff, people with dementia and family carers, and comparative case studies).

## Results

The integrative analysis identified seven key factors which influence the delivery of good EoLC for people with dementia (Table 2).

**Table 2:** Summary of the seven factors influencing good EoLC for people with dementia

Undertaking timely planning discussions to ensure plans are discussed when the person with dementia has capacity and that they are documented and disseminated as appropriate.	
Recognising end of life and providing supportive care to ensure effective management of key symptoms (e.g. pain, anxiety and nausea), and minimise distress by providing comfort in a familiar environment.	
Co-ordination and continuity of care includes liaison between day and night staff in services and having established links with local services (e.g. hospices), particularly for support out of hours.	
Working effectively with primary care can be facilitated by having a named liaison person in the practice. For care homes, liaison can be improved by regular routine visits and limiting the number of general practices with which residents are registered.	
Managing hospitalisation includes avoiding unnecessary admissions by appropriate out-of-hours support and documentation of wishes and preferences. It also involves managing admission and discharge effectively where hospitalisation is necessary.	
Continuing care after death to enable family members to be supported by known members of staff who cared for the person with dementia at the end of life. This continuity of care is valued by family members.	
Valuing staff and ongoing learning facilitates staff retention and results in a more skilled and knowledgeable workforce. Stable staff teams are more able to detect emotional vulnerability in their colleagues and ensure timely and appropriate support.	

While working effectively with primary care and managing hospitalisation clearly relate to co-ordination and continuity of care, we have chosen to present them as separate factors owing to the extensive discussions of these particular issues. Each factor is described below including the differences in emphases and priorities found within and between stakeholder groups.

### Timely planning discussions

National experts and service managers viewed timely planning discussions as key to meeting the preferences and wishes of people with dementia at the end of life:

*But also, if there hasn’t been discussions with relatives, everybody becomes anxious at that point, and that’s why putting all these things in place is quite significant, so that you are not ending up in a situation where you are in the middle of a crisis, and you are having to make rash decisions. You are just acting and activating whatever you’ve already set in plan. (SM11, service manager, specialist EMI care home)*

Finding the ‘right’ time for such discussions was, however, problematic; the point of diagnosis was seen as too early, but other opportunities often did not arise until the person with dementia had relatively severe impairment (e.g. on admission to a care home). There were also tensions over responsibilities and skills for timely planning discussions. Our data suggest that the majority of such discussions involved general practitioners (GPs), senior nurses and/or service managers, all of whom had established relationships with patients and carers and could adopt an iterative approach to exploring preferences:

*There’s not an easy way to approach it, but it’s little by little. Generally they’ll start off with a conversation if they’ve been having a lot of infections, ‘so how do you feel about this?’ And gradually get into it, whether it’s over a course of a couple of hours whilst they’re in [the home] or maybe a couple of weeks, just building up to the conversation, easing them into it. (FS16, nurse, staff focus group, specialist EMI service 2)*

Staff with ongoing relationships were also able to explicitly address and manage differing views within families:

*With regard to families who maybe don’t agree with each other or don’t get on, it is knowing how to support the family to talk to each other and come to some sort of agreement – it is seeing the bigger picture and talking to the family and supporting them to put the thoughts of the resident first, then backing away. Certain relatives may get on better with certain staff so it’s working as a team to deal with that. (Fieldnotes of informal discussion with FS03, senior care assistant/team lead, supported living service 2, 30.9.15)*

Some professionals expressed concerns over the quality of planning discussions conducted by care home staff. It was clear through observation and the ways staff described their conversations about future plans that discussions often involved family members rather than people with dementia. Even following such discussions, family members were not always clear whether plans had been made, nor on the content of such plans. Despite these concerns palliative care or hospice staff with specific expertise in advance care planning were rarely involved in such discussions or in supporting staff who were undertaking them.

Discussions with people with dementia highlighted a range of barriers to planning ahead including: a preference to focus on living in the present; a lack of awareness that dementia is a terminal condition; assumptions that family members and health care professionals would know their wishes and be able to make decisions on their behalf if necessary; confidence in the quality of current and future service provision; and difficulties in engaging in discussions about end of life at a time when they felt fit and healthy:

*Well you can’t plan - for something in place when you don’t know when you’re going to die. (PWD16)*

Family carers similarly reported some tensions in discussing EoLC: many were not aware that dementia was a terminal illness; others preferred to focus on the present; and for some, such discussions implied that they were eager for the death of the person they supported. The value of discussing EoLC was, however, often more apparent to carers faced with making decisions towards the end of life. At this point, many carers realised that they were uncertain about the wishes and preferences of the person with dementia and could find decision-making burdensome:

*I mean I’ve known him since I was 18, and I’m 78 now, and you would think I would have known his wishes but it’s a thing he never ever, ever, talked about. […] we never ever talked about dying. Now I wish we had done, it’s a funny thing you know, it is because if me and (husband) had discussed it I would say “right I’m doing his wishes” but now I don’t know. (CC07, care home 1)*

When people with dementia and carers had approached planning independently with no professional input, they were often unaware that certain decisions could only be made by medically qualified professionals (e.g. ‘do no attempt cardiopulmonary resuscitation’) or that plans needed to be documented and disseminated appropriately if they were to be followed.

### Recognition of end of life and provision of supportive care

While people with dementia and their families did not discuss recognition of the end of life, professionals expressed diverse views on this issue. Many national experts viewed the identification of end of life in dementia as problematic and hospice staff similarly emphasised the uncertainty of the dying trajectory in dementia. Several care homes adopted proactive approaches to identify residents approaching the end of life. These included the Gold Standards Framework [35], a UK initiative developed to improve primary care-delivered palliative care, discussion of residents at monthly meetings with a palliative care team, and review of measures such as weight and functional ability. Experienced professionals, however, often relied on subtle shifts in behaviour, or physical changes as indicators that the person might be approaching the end of life:

*“He always used to have a beer at mealtimes. He stopped asking for any of that, you would offer him, he didn’t want it anymore, so we could see that things with [resident name] were beginning to turn.” (Case study interview with SM11, service manager, specialist EMI service 1, 28.7.2015)*

Actions triggered by the recognition that a person with dementia might be approaching the end of life included simplification of medication and the addition of new planning documents (e.g. regarding resuscitation, hospitalisation and emergency healthcare plans) as needed. In addition, discussions about the end of life were typically initiated with carers to clarify expectations, their preferred level of involvement, frequency and timing of updates and to review existing end of life plans. Bereaved carers generally felt that they had been informed that end of life was approaching suggesting that these discussions were successful.

Aspects of supportive care at the end of life included: ensuring that the person was comfortable and pain free; meeting psychological and spiritual needs of people with dementia and their families; facilitating family involvement; and creating a peaceful environment. All stakeholder groups agreed that the end of life should be pain free and comfortable:

*“I’ve seen people with, suffering with cancer, being in a lot, a lot of pain and, I don’t think that’s right somehow or other, I never felt it was right for them I don’t think it’s right for me either. So I think the pain relief, pain management, one of the most important things when you’re getting towards the end. It my own opinion, I’d rather have the pain decrease, even if it meant that I was going to live shorter.” (PWD26)*

While some national experts expressed concern about pain management, service managers and frontline staff were confident that their knowledge of the individual and understanding of dementia enabled them to recognise non-verbal signs of pain. Access to anticipatory medications enabled staff to respond quickly if people with dementia exhibited new symptoms such as anxiety and nausea or required stronger pain relief but was sometimes problematic (see section on Effective working relationships with primary care).

The need to address emotional, spiritual and cultural beliefs was emphasised by all stakeholder groups. Emotional support to both people with dementia and carers could be provided through simple physical acts such as holding a person’s hand or sitting with the person with dementia and carers to reassure and comfort them as needed:

*“I just think it must be really scary and frightening. You just don’t know what they are thinking. Even if you are there it is just reassurance. I know myself when I have gone in there, it is speaking to people and saying, ‘Are you alright? I am here.’ You just don’t know. At least you can go away knowing you may have been there or provided a little bit of comfort while they are passing. It just gives you job satisfaction. Like I say it is the last thing you will ever do for them. They are leaving this world and you would like to think you have maybe made a difference to their last moments.” (Case study interview, FS77, night care assistant, specialist EMI service 1, 24.9.2015)*

Spiritual support often centred round religious practices. Tensions between staff arose where wishes and preferences regarding aspects of care were not followed, for example when some staff (often agency) imposed their own values (e.g. praying in the room of a person with dementia with no religious beliefs). This failure to respect the wishes of people with dementia and their families undermined the efforts of those staff members who were closely involved and strongly invested in providing good EoLC.

Facilitating family involvement was seen as important by all stakeholders and started with conversations that the person was approaching the end of life. The need to support families to say ‘goodbye’ in their own way was also emphasised:

*“They actually had a party with their mum a couple of nights before she died, it was just before mother’s day, they were all absolutely gone on vodka, and mum had a couple of Baileys just before she died, so it’s about making that family environment comfortable for the families I think is important.” (SM14, manager, care home)*

Where the end of life was protracted or involved a series of ‘false alarms’, ensuring families had sufficient support was essential:

*“People can sustain looking after somebody at the end of life for a few days but if it goes on it gets very difficult […] families get mentally and physically exhausted unless there’s a big family. They just get worn out. So then they get stressed, then they fall out with each other and all the tension rises.” (SM22, service development lead, supported living service 1)*

Creating a peaceful environment towards the end of life was emphasised by professionals but was discussed less frequently by people with dementia and their families. Many professionals discussed the importance of a peaceful environment, for example, through soft lighting, flowers, photographs or treasured possessions, emphasising the importance of tailoring this to the individual:

*“What sort of ambience are you trying to create in that particular room setting? Are you involving music, are you involving tactile things such as hand massage and keeping them calm and speaking quietly, but you’ll need to know the people’s life story to understand what they would like, especially in relation to music.” (SM13, owner/manager, home care service)*

While people with dementia valued a homely environment and objects of personal significance, this was not specific to end of life but was relevant to any transitions during the illness trajectory. Current and bereaved carers rarely commented on the physical environment and the extent to which they valued or noticed staff attempts to create a peaceful environment was unclear.

### Co-ordination and continuity of care

All stakeholders agreed that people with dementia should die in their usual place of care whenever possible; this required close coordination between different agencies:

*“The best end of life care is one that involves a joint partnership working, district nurse team, family, home. You can actually really support people to die well if that partnership is working really well, and the GP.” (SM06, service manager, care home)*

However, all stakeholder groups (except people with dementia) described some difficulties and tensions in ensuring appropriate support at the end of life. Carers were often responsible for co-ordinating different services at the end of life when people died at home and could find this challenging because of the lack of communication and shared patient records between services:

*“The hardest part is actually trying – because you end up the hub at the middle that’s trying, in areas that you don’t know what you’re doing anyway, but trying to get it all to fit together.” (BC02, supported living service 1)*

To relieve the burden on family carers, national experts highlighted the need for one professional to be responsible for clarifying pathways and co-ordinating care. This could also facilitate access to continuing health care funding to support EoLC in people’s own homes. The increased care needs at end of life could create discontinuity if existing services did not have the capacity to provide the level of input required or if a care home resident had to move to access nursing care. Flexibility of working arrangements could mitigate these problems and avoid changes in personnel at the end of life:

*“I think families appreciate that [staff working extra shifts]. In this sad time the families appreciate that they’re not having a stranger coming in, thinking ‘who is this coming in to deal with my mam’s personal care?’ They know exactly who is coming in.” (FS31, senior care assistant/team lead, staff focus group, supported living service 1)*

Local hospices and/or palliative care teams tended to be used to support generalist staff, through advice and/or formal training, rather than being directly involved in providing EoLC to people with dementia. While hospices often aspired to greater involvement in dementia care, their role seemed unclear and their staff uncertain about engaging with this patient group. A focused outreach model was thought to be most appropriate in dementia, with an emphasis on upskilling generalist staff:

*“I’m not sure that we actually need a specialist palliative care in the way that we have specialist palliative care in cancer care. I think we need a more distributed model, so that, for example, psychiatrists for older people and community psychiatric nurses have some of the those specialist skills in palliative care, and that they can employ them in the community, rather than waiting to refer on to some sort of specialist group.” (NE16, academic palliative care)*

Most care home staff were keen to provide EoLC for residents and saw this as an important part of their role. A number of barriers, however, were identified including access to support with setting up syringe drivers, and inconsistent access to anticipatory medicines.

Co-ordination *within* as well as between services was also emphasised, with effective liaison between day and night staff being problematic in some care homes. Residents seemed to die more frequently during the night, yet our data suggest that night staff may be less well placed to deliver good EoLC. During night shifts, staffing levels are lower, typically with only one nurse on duty. This minimises opportunities to discuss management and to share decision-making if any problems arise. Furthermore since night staff typically were less familiar with the person with dementia and family, they sometimes seemed to act on their own values and preferences rather than adhering to wishes recorded in the notes:

*The family did not want to be contacted in the middle of the night should anything happen, and this was well documented in notes/files and reported at the staff handover. However, the family were called in the early hours of the morning [by an agency nurse] to be informed of the death. (Fieldnotes, care home 1, 12.10.15)*

Tensions in collaborative working could arise where the detailed knowledge and expertise of care staff was not acknowledged or valued by external professionals.

### Effective working relationships with primary care

The key role of primary care in EoLC in dementia was recognised by all stakeholders, though not explicitly by people with dementia. GPs were identified by national experts as the most appropriate care providers for ‘uncomplicated’ end of life for people with dementia:

*“I think that for a lot of people with dementia, they don’t actually need anything more than that, in the sense that they haven’t got any difficult behaviours, they haven’t got depression, they haven’t got delirium, they’re not in terrible pain, so they don’t need that specialist palliative care input. They need very basic terminal care and it’s completely within the remit of general practice to provide that.” (NE31, academic & clinician, dementia and palliative care)*

National experts and carers of people with dementia in care homes emphasised the importance of opportunities for carers to discuss end of life (and other concerns) directly with GPs to ensure that they were well informed and actively involved in decision making. Carers of people with dementia dying in their own homes highlighted the key role of district nurses in providing practical support and equipment to facilitate EoLC:

*“I mean they were very good in as much we got a bed, they saw that we not only got a bed but one of these water mattresses, everything was in place.” (BC01, supported living service 1)*

Effective working relationships between care homes and the primary care team were facilitated by aligning a single GP practice with a care home and by having a named practice contact (practice nurse or GP) and/or weekly ‘ward rounds’ in the care home. The latter could be used for regular reviews of all residents to ensure a proactive approach in which minor issues were addressed and any signs that the person might be approaching end of life could be discussed. In contrast, care home staff found it difficult to manage relationships with multiple GPs and practices. Their difficulties were exacerbated by the variation between individual GPs in terms of their skills and interest in working with people with dementia; extent to which they recognised and valued the expertise of care home staff; and attitudes to anticipatory prescribing:

*“We’ve one GP practice we get absolutely superb support and care, it’s very, very integrated so whatever the GP does it’s done in partnership with the care home and it’s done with family involvement and, if appropriate and relevant, with the individual themselves. With another GP practice it’s all instigated by us with very little input from the GP and very little support and very little partnership.” (SM03, senior manager/director, care home)*

One care home had successfully developed and agreed processes, responsibilities and documentation for EoLC with local practices to address inconsistencies in anticipatory prescribing.

Out of hours primary care support was consistently identified as an area for improvement by service managers and frontline staff with shortcomings of existing services seen as contributing to unnecessary hospitalisation at the end of life. While specialist hospice or palliative care services were available out of hours, their use seemed inconsistent.

### Managing hospitalisation

Most stakeholders emphasised the need to avoid unnecessary hospitalisation towards the end of life. While people with dementia were confident that hospital staff would have specialist skills in dementia care, many participants described significant shortcomings in inpatient care. Even with good quality care, the process of admission could be disorientating and distressing for people with dementia. While people with dementia did not specifically discuss their preferences regarding hospitalisation, their emphasis on continuity of care and the desire to stay in their own home for as long as possible suggests that they would prefer to avoid hospitalisation. Carers were confident that the person they supported would wish to avoid hospitalisation and die in their usual place of care if possible:

*“I sort of agreed with everybody here and the GP that if at all possible there would be no hospitalisation again because I personally felt she got far better care here, than she would get in hospital. I mean I’m not criticising the hospital, I just think places like this are better set up for caring than a hospital ward is and it seemed to me she was far less disturbed by being here with the people around her that, she kind of recognised.” (BC09, supported living service 2)*

Documenting patient preferences regarding hospitalisation was identified as an important way of avoiding unnecessary admission. Where such documents were shared with OOH and ambulance services, staff reported being more confident that they could seek advice without risking admission. National experts and some service managers attributed some admissions to a lack of staff confidence and limited support for night staff in care homes. While one service manager had tried to circumvent these problems by encouraging night staff to contact senior members of the care team before involving the OOH service, the extent to which this had been successful was unclear. Increasing clarity over the purpose of involving the OOH service was identified as a potential strategy for avoiding unnecessary hospitalisation. Since the alternative to calling the OOH service was to ‘do nothing’, national experts suggested reframing this option as a positive action which would enable the person to remain in their preferred place of care:

*“Care staff want to do something…the biggest benefit for the patient is not to send them to hospital, that will only cause harm. To have the confidence to see that in a very positive reframe, if you like, “That I’m really doing something really important here, by keeping them here” I think is fundamental.” (NE29, clinician, palliative care)*

Where admissions were unavoidable, professionals emphasised the importance of providing hospital staff with information to enable them to provide person-centred care, although some care staff doubted whether such information was used by hospital staff. Professionals and carers also stressed the importance of rapid discharge to minimise the negative outcomes associated with hospitalisation. Improved discharge planning could reduce length of stay and ensure that any equipment or medication was available on return to the usual place of care:

*“I don’t think that [hospitalisation] is a problem as much now. It used to be, but I think we’ve built up a good relationship with [name of hospital] as well, and we can phone them up and say, ‘Look, can this person be rehydrated but then come home?’” (FS04, service development lead, staff focus group, supported living service 2)*

All professional stakeholder groups acknowledged that the delivery of good EoLC was compromised when patients were discharged from hospital to a new care home specifically for EoLC. Unless a family member was immediately involved, care staff often had little information about the patient, since discharge notes and medication did not necessarily arrive with the patient and there was typically a delay in access to primary care records.

### Continuing care after death

Service managers and frontline staff described the ways in which the person with dementia was cared for in the period between death and removal from their usual place of care. This typically included ‘last offices’ where the person was washed and dressed:

*They usually dress the person in a clean nightie or pyjamas and leave them lying on the bed. They always pick some flowers from the garden and place these on the pillow by the person’s head. Although the undertakers may wrap the person in a sheet before transporting them, care staff just leave the person as though they were lying in bed. (Fieldnotes of informal discussion with FS33, nurse, specialist EMI service 1, 24.7.2015)*

These activities were an important part of EoLC and often gave staff a sense of satisfaction and closure. Another aspect of continuing care after death discussed by all stakeholders was the provision of post-bereavement support for carers. This was seen as an integral part of EoLC by professionals. People with dementia expressed a desire for their families to receive support if needed:

*“I would just want the people I was leaving behind to be helped and taken care of and anything that could be done for them done.” (PWD21)*

Carers often welcomed practical support immediately following the death, for example, help with contacting family members or liaising with the funeral director. Many carers also valued the attendance of staff at the funeral:

*“I’m her only relative, apart, then I’ve got two children and their wives, so I thought, ‘there’s only going be six of us at the funeral’ and I thought ‘oh, it’ll look horrible’, and they all turned up from the home.” (BC12, supported living service 2)*

Although following the death of a person with dementia there was often no formal reason for the carer to maintain contact with the service, staff emphasised the importance of continued support at least in the short term:

*“Because the residents have got elderly, their families tend to be elderly and their whole sense of purpose has been around visiting an elderly relative in a nursing home now once they’ve died, they’ve then got a lack of purpose. So sometimes we’ll see like for the first six months they’ll come in quite regularly cause that’s part and parcel of their routine and then hopefully what we hope to see is it just tailing off because then we know that they’re getting on with life.” (SM28, service manager, care home)*

Some service managers arranged an informal follow-up contact around 6 weeks following bereavement to gauge how the carer was coping and signpost them to specialist bereavement services if required. The interviews with bereaved carers highlighted the value they placed on this continued care and support from professionals.

### Valuing staff & on-going learning

Much of the data for this theme relates to care staff working in care homes or community services. The need to value staff was not expressed in relation to clinicians or other care professionals. Clear training needs, however, were identified for GPs and hospice staff. In particular, senior staff in a number of different care homes reported that some GPs had negative attitudes towards working with people with dementia and lacked skills in communicating effectively with this patient group:

*“I just think a massive thing is GP awareness […] if somebody could cure that with GPs I think people living with dementia will have such a different, different life.” (FS07, manager, staff focus group, supported living service 2)*

A number of senior care staff, including nurses, described how they tried to change GPs’ behaviour by modelling good communication and encouraging face-to-face contact with residents. The reluctance of some frontline staff in hospices to extend their services to people with dementia, despite the desire to do so among senior staff, also suggests a need for training:

*“I think there is a stigma to dementia and I think palliative care thinks it’s probably a bit swanky and, you know, we do cancer, we’re just starting to do lung and cardiac and renal disease and a bit of neurological disease. I think there’s an element of ‘it’s not what we do’.” (SM35, service development lead, specialist palliative care service)*

The challenges of working in the care sector were acknowledged by all stakeholder groups. Since good EoLC required detailed knowledge of individual people with dementia, a stable workforce was essential. While the capacity to work flexibly and increase staffing levels if needed towards the end of life was recognised, achieving this in the context of limited resources could be problematic. In a sector with limited scope for promotion or improved pay, some stakeholders emphasised the importance of leadership:

*“One care home will be brilliant and another care home would be disastrous and that’s about leadership of the care home and it’s about valuing the staff. If the staff are not valued they feel very taxed about caring for the psychological needs and behavioural needs of people with advanced dementia because, it’s tough and if they’re not valued for the work that they do and management is aloof and doesn’t understand the difficulties and the shortage of staff, there will be difficulties and it won’t be a nice place to be cared for.” (NE36, academic/clinician, dementia and palliative care)*

Alternative approaches to valuing staff were also suggested, including training opportunities and the creation of specialist roles (e.g. ‘end of life champion’). Investment in training varied significantly between care homes; some homes had developed their own training packages and had dedicated trainers; others adopted a cascade model of training; while other care homes seemed to have a more ad hoc approach to training focused on meeting minimum standards. Homes which prioritised training incorporated it as part of the ‘normal’ working day; in others, staff were sometimes expected to attend training in their own time.

The personal qualities of staff were identified as important by all stakeholder groups. People with dementia and carers valued attributes such as kindness and compassion and appreciated staff who ‘go that extra mile’. For some services with a strong person-centred ethos, values and qualities were of primary importance and technical skills were developed through in-house training.

The issue of attachment between staff and people with dementia was most frequently raised by service managers and frontline staff but was also highlighted during the observation and commented on by some carers:

*“They liked him, they loved him even, they were fond of him and they cared for him as somebody they were fond of. They responded to his needs.” (BC003, supported living service 1)*

While some service managers explicitly advised staff not to become attached to people with dementia, others saw emotional attachment as an essential part of the job. The observation highlighted the emotional work involved in providing EoLC, particularly where the development of close relationships with residents was prioritised. Despite loss being a recurrent event for staff, emotional support for staff tended to be informal across all settings. This seemed to work most effectively where staff worked in small, stable teams which enabled them to identify early signs of emotional vulnerability in their colleagues and ensure that appropriate support was put in place:

*“She [team lead] made us feel comfortable by saying, ‘You’ve done your best for that lady now. She’s on her journey now and you’ve carried her through it.’ And that made us feel better you know and we knew then that she was happy and we had done our best for her.” (Case study interview, FS55, care assistant, supported living service 2, 21.10.15)*

In care homes where staff worked across the home rather than with one specific group of residents, observation suggested that informal support often centred on established staff groups with new members of staff sometimes being marginalised. Managing personal bereavement whilst working with people at the end of life was identified as particularly challenging for staff and an area in which little formal support was available.

While training on EoLC was often available to care home staff (often provided by hospices and palliative care teams) opportunities to learn from experience were also important. Senior staff were often thoughtful when introducing new staff to EoLC, encouraging them to become involved at their own pace. Tensions arose in one care home, however, where members of staff with a particular interest in EoLC wanted to be involved in all deaths, limiting opportunities for new staff to engage in EoLC. Reflective post death meetings which encouraged staff to reflect on the care provided to identify positive aspects and any implications for improving care were not widely used.

## Discussion

This paper reports one of the largest empirical studies of EoLC in dementia which used a range of methods to assimilate evidence from different stakeholder groups, including people with dementia. Our findings provide detailed insight into factors which influence the provision of good EoLC and demonstrate that these factors are inextricably linked so that deficiencies in one area undermine the extent to which other aspects can be achieved. Some areas were more important to professionals (national experts, service managers and frontline staff) than to people with dementia and their families, for example future care planning and recognition of the end of life phase. As would be expected, national experts generally had a more strategic view than other stakeholder groups, who tended to focus on the practical day-to-day aspects of EoLC provision. Many of the challenges we identified in delivering good quality EoLC to people with dementia have been highlighted in previous research [13, 20, 22]. However, where our study adds considerably to the literature is in integrating the views of a range of stakeholders and highlighting areas of consensus and of differing priorities.

The study also provides empirical data to inform policy and practice guidance [17, 36–38] which to date has largely relied on professional opinion. In comparing our findings with such guidance [17, 36, 38], there is considerable agreement with the areas of care identified for ‘optimal delivery’, such as future care planning and co-ordination of care. However, UK guidance neglects to include the need to support and train professional staff [39]. It also represents optimal care as a linear process with six steps, whereas our data indicate that a more cyclical ongoing process with constant review of the seven key themes is required. Our seven themes are consistent with the 11 domains for optimal EoLC in European Guidelines [17] but specifically highlight the importance of primary care. This may reflect the fact that in the UK, the majority of EoLC to people with dementia is provided by community-based services. Several domains identified in the European Guidelines were subsumed within our theme of recognising end of life and providing supportive care (e.g. spiritual support, ethical issues and the creation of an aesthetic, safe and supportive environment). People with dementia and family carers, however, rarely identified factors relating to the physical environment as important to the provision of EoLC, instead prioritising the interpersonal aspects of care. This raises the question of whether providing a peaceful environment at the end of life (with music, flowers, photographs etc.) is more for the benefit of staff (providing them with a sense of being able to ‘do’ something) than for the person with dementia and their family.

Advance care planning in dementia continues to be emphasised in policy and considered a professional marker of good quality care, although further high quality studies are required to provide robust evidence of its clinical and cost effectiveness [40–43]. The lower priority placed on future care planning discussions by people with dementia and their families is consistent with previous studies [44–46]. Many people with dementia and families prefer to live in the present and struggle with the concept of planning ahead for their future care. Our findings suggest that possible strategies for increasing engagement in such discussions may be to frame such discussions in terms of: reducing future stress for family members, enabling family members to act in accordance with the wishes and preferences of the person with dementia; or preventing unnecessary hospital admissions (particularly in light of a recent study which showed that people with dementia were four times more likely to die comfortably if they remain in their ‘usual place of care’ [47]). Other authors have suggested that adopting more informal approaches to ACP may be more acceptable to patients and carers [48]. Regardless of how such discussions are approached, access to staff with specific expertise in undertaking such sensitive discussions may also be needed as many professionals, regardless of speciality, lack the confidence and/or skills to do so [49]. Furthermore, strategies are needed to ensure that the outcomes of such discussions are accurately documented and disseminated [50].

Consistent with existing literature, professionals with little involvement in the day-to-day care of people with dementia (primarily national experts and service managers based in hospices) emphasised the difficulties in identifying end of life in dementia and the uncertain trajectory [51, 52]. However, frontline staff and service managers responsible for delivering end of life care, seemed confident in their ability to recognise subtle changes indicating that the end of life might be approaching and accepted that the trajectory might not be linear, but could be characterised by periods of recovery and deterioration. From the patient and family perspective, it seemed more important for professionals to provide person-centred, supportive care throughout the illness, rather than struggle with the dilemma of when to move to a palliative care approach [44, 45, 53]. Our findings appear to add weight to the argument favouring use of the term supportive care, rather than palliative care, in dementia as such a phrase would be applicable throughout the whole illness trajectory [54] without evoking the common perception that palliative care refers only to terminal care [55].

The emphasis on training and valuing staff in our work is consistent with previous research which has emphasised the need to upskill staff [19, 56, 57]. A number of studies have highlighted the lack of recognition of the knowledge and expertise of non-qualified care staff who are most closely involved in delivering care [58, 59] and the implications this can have for the provision of care [60]. Previous studies have also emphasised the need for person-centred management and interventions to meet the emotional support needs of care home staff [61–63]. Effective co-ordination of care requires an emphasis to both effective working relationships and improved communication across organisational boundaries [22, 64, 65]. While specialist services, such as palliative care teams and hospices, sometimes had a key role in supporting and upskilling existing staff to enable them to care for people with dementia at the end of life, consistent with previous research, concerns over capacity, skills and knowledge of dementia were identified [56].

Supplementing the interview and focus group data with case studies provides new insights into the realities of providing EoLC for people with dementia. By deepening our understanding of the processes, routines and skills required to ensure a ‘good death’, the data complemented the information gathered from stakeholders. While we aimed to include the experiences of people dying with dementia (i.e. dying from an intercurrent illness rather than from advanced dementia) and those dying in their own homes, the data focused primarily on those living and dying in care homes. Although this reflects the experience of many people with dementia and carers, with over half of people with dementia in the UK dying in care homes [3, 66], a broader representation of the range of end of life experiences would have increased our confidence in the relevance of our findings to a range of settings. It proved difficult, however, to engage domiciliary services particularly in the observational component of our work.

The seven key themes emerging from our data are core to both the provision of good quality palliative care in general and also to the principles of long term illness management; they are however challenging to deliver because of the complexity of the systems involved. The question remains as to how best to translate these findings into practice. Other studies have suggested the introduction of new roles such as a mobile specialist palliative care team in dementia to provide expert advice and to support the usual care-giving team [28], or a ‘key worker’ to co-ordinate care [50]. A number of innovative options to improve EoLC in dementia have been tested, including dementia specific hospice care [67]; specialist dementia palliative care [68]; a specialist community-based multi-disciplinary team focused on facilitating home death [69]; an ‘integrated care leader’ [70]; audit/feedback systems [71], decision support tools [72, 73] and a multifaceted intervention in long term care [74]. Such studies are, however, often small scale and lack rigorous cost effective evaluation to support wider implementation. The next challenge for this study will be to translate our key findings into a practical intervention and test its feasibility and acceptability in usual care settings before proceeding to determine its effectiveness and cost effectiveness compared to other models.

## Conclusions

This unique study provides a rich evidence-base which confirms the relevance of much of the content of existing end of life frameworks to dementia. It also highlights the different priorities which characterise different stakeholder groups, drawing attention to the need to tailor interventions to individual people with dementia and their carers. The findings indicate that a wide range of areas need to be addressed by initiatives to improve EoLC in dementia. In addition to addressing all seven of the areas suggested by our research (rather than focusing on specific aspects in isolation), any intervention needs to focus both on optimising care delivery to individual people with dementia as well as their families and on developing systems in terms of policies and procedures to support EoLC.

## Additional file

Additional file 1: Description of case study sites. (DOCX 17 kb)

## Abbreviation

EoLC: End of life care. This is care that: “Helps all those with advanced, progressive, incurable illness to live as well as possible until they die. It enables the supportive and palliative care needs of both patient and family to be identified and met throughout the last phase of life and into bereavement. It includes management of pain and other symptoms and provision of psychological, social, spiritual and practical support” [75].
