# Supplementary material for: What enables good end of life care for people with dementia? A multi-method qualitative study with key stakeholders
Source: BMC Geriatr. 2018 Dec 4;18:302. doi: 10.1186/s12877-018-0983-0 (PMC6280541; doi:10.1186/s12877-018-0983-0)
Supplement: Supplementary file 1 — Description of case study sites. (DOCX 17 kb) [file 12877_2018_983_MOESM1_ESM.docx]

# **Additional file 1 – Description of services in comparative case studies**

## **Care home – ‘usual care’**

The care home is one of a family owned care group of 12 homes and is located within a suburban area. The home is for people with a history of mental health problems or a diagnosis of dementia. It is a purpose built care home with 44 single rooms (11 ensuite), split over three floors with residents separated by gender. Each floor has an almost identical layout with a nurses’ station easily accessible to all residents. Staff used to be allocated to a specific floor but the new manager (who came into post shortly before fieldwork began) introduced a more flexible system where staff are allocated to a floor at the beginning of their shift. Staff views on the changes varied; some preferred the previous system which enabled them to develop good relationships with residents and their families (and also gave a sense of ownership over the floor, with one member of staff describing the floor on which she had previously been based as ‘my home’); other staff could see the advantages of the new approach:

*The two care workers felt this worked better as in the past if they had to cover another floor, they did not know the residents and their needs and described how it felt like their first day. They also felt that they didn’t know where things were on other floors, and how each ‘floor’ liked to be run. They felt there might be a bit of trade-off between knowing people really well, but felt they got to know most of the residents over time. (Fieldnotes, care home 1, 24.6.15)*

There are no allocated meeting rooms in the building so the downstairs dining room is often used for this purpose. The staff room was used for storage and staff breaks were often taken in the dining room on the floor working on or outside in the garden. There were several changes in the senior management team during the process of negotiating and conducting the fieldwork.

## **Specialist EMI service – ‘good practice’**

This specialist EMI home is located in a rural area and provided by a leading global health and care company. The care home is a converted house which has undergone a series of transformations, including a refurbishment during the period of observation. The home has 55 single rooms, most with ensuite facilities. All resident rooms and communal areas are on the ground floor, with only the staff room, manager and administrative offices based upstairs. The nurses’ station is situated at reception and is not accessible to residents. The majority of residents had fairly advanced dementia, with many having been transferred from other care homes where their behavioural and psychological symptoms could not be managed. While the home has several distinct lounges, each with an attached dining area, there are no physical boundaries between areas within the home so that residents can move between the lounges. The following extract from fieldnotes describes the difficulties experienced by one of the researchers in navigating the site:

*Interestingly, I was much more disoriented today than I had been the previous day […] Given how totally lost I kept getting, the residents don’t stand a chance. I have absolutely no mental map of the layout; it just seems completely confusing. (Fieldnotes, specialist EMI service 1, 17.7.15)*

Staff in Field View work across all areas of the home with no formal staff teams. Day staff were allocated to a specific area for the morning shift and after lunch were reconfigured and allocated to new areas, night staff worked as a single team across the entire home. While the home had a stable core team, there was considerable turnover amongst care assistants, reflecting the challenging environment within this home.

## **Supported living service – ‘good practice’**

This is part of a pioneering approach to care from a longstanding not-for-profit care service organisation. Residents require personal, nursing or dementia care. Based in a suburban area it is a specially designed community complex with six supported households, each with either ten or twelve ensuite rooms and open plan living spaces including a kitchen area where meals are prepared. Staff and residents often eat together in the households and there is also a bistro within the complex that is regularly used by staff, residents and families. The aim is to create a family atmosphere and staff work in small teams within their own designated household. The nurses move around all of the households and have use of a computer room for their paperwork:

*We are a close-knit team aren’t we, they are small households; so we are close aren’t we? We are like a family. (FS03, senior support worker, staff focus group, supported living service 2)*

There are a number of other spaces available in the complex including a gym, hairdressers, function room with bar, internet café and meeting rooms. The service also has 12 self-contained apartments as part of the community. This home was staffed by a stable senior management team and a large core workforce; staff turnover was either due to a change in career or a lack of ‘fit’ with the ethos of the service.
